# A compiled dataset of the energy usage indicators and unit energy consumption values available in Ireland

**DOI:** 10.1016/j.dib.2021.107204

**Published:** 2021-06-07

**Authors:** Connor McGookin, Brian Ó. Gallachóir, Edmond Byrne

**Affiliations:** a Energy and Policy Modelling Group, MaREI Centre, Environmental Research Institute, University College Cork, Ireland; b School of Engineering, University College Cork, Ireland

**Keywords:** Local energy planning, Regional energy transitions, Municipal energy planning, Sustainable energy action plans, Covenant of Mayors

## Abstract

This dataset supports the analysis outlined in (McGookin et al., 2021) “*An innovative approach for estimating energy demand and supply to inform local energy transitions*” [1]. It consists of four key elements: a range of different energy usage indicators (e.g. the number of employees or cars in a region), national unit energy consumption values, energy supply fuel shares per sector, and an overview of the housing stock. Firstly, the range of socio-economic statistics used as indicators of energy demand are primarily gathered from the Central Statistics Office's (CSO), along with sector specific sources like the Department of Transport or Fisheries. Secondly, the national energy demand and supply in the five sectors of agriculture and fishing, industry, residential, services and transport comes from the national reporting body Sustainable Energy Authority of Ireland. These two datasets are then used to form the national unit consumption figures for a range of indicators in each sector. A national unit consumption value gives an average energy demand per statistical unit, for example MWh/employee or MWh/km driven. This can be used to estimate subnational energy demand in the absence of recorded energy data below the national level. Finally, the Building Energy Rating database (which is reported quarterly by the CSO) provides details on the Irish housing stock and non-domestic building's primary heating fuels.

## Specifications Table

**Table:** 

Subject	Engineering (General)
Specific subject area	Developing an estimate of subnational energy demand and supply
Type of data	Tables
How data were acquired	• Census data gathered from central statistics office (CSO) small area statistics portal - http://census.cso.ie/sapmap/ • Business and transport surveys taken from the Central Statistics Office data portal - https://data.cso.ie/ • National energy balance taken from Sustainable Energy Authority of Ireland reporting - https://www.seai.ie/data-and-insights/seai-statistics/key-publications/national-energy-balance/ • The Building Energy Rating database is published quarterly by the central statistics office, with a series of tables available for download as a .csv file • Irish Bulletin of Vehicle and Driver Statistics published annually by Department of Transport • Irish Sea Fishing Fleet Register published annually by Department of Agriculture, Food and the Marine
Data format	• Raw input data • Filtered input data
Parameters for data collection	• Range of socio-economic values that may be used as indicators of energy demand such as employees or distance travelled by cars • Energy statistics for Ireland covering the energy demand and supply in the five sectors of agriculture and fishing, industry, residential, services and transport • Housing stock data covering the energy demand and size by age • Primary heating fuel supply for non-domestic buildings
Description of data collection	• All data from the CSO is available for download as a .csv file, and was filtered as outlined in Section 2 • The national energy statistics are taken from annual reports prepared by Sustainable Energy Authority of Ireland • Other data taken from government reports from Department of Transport and Department of Agriculture, Food and the Marine
Data source location	The data covers national energy and socio-economic statistics for the country of Ireland, as well as two subnational regions within Ireland; County Kerry and the Dingle Peninsula.
Data accessibility	Repository name: Mendeley Data Data identification number: https://doi.org/10.17632/rttsn7ptxk.1 Direct URL to data: https://data.mendeley.com/datasets/rttsn7ptxk/1
Related research article	C. McGookin, B. Ó Gallachóir & E. Byrne. ``An innovative approach for estimating energy demand and supply to inform local energy transitions.'' Energy (2021): 120731. https://doi.org/10.1016/j.energy.2021.120731[1]

## Value of the Data

• Making the data available is important to ensure the research is transparent and accessible.
• Energy planners seeking to understand the current energy demand and supply in their region, city or town will be able to see the energy usage indicators used in this study.
• Comparing this data against other countries would give valuable insights into the representativeness of the various energy usage indicators and varying energy profiles of different countries.

## 1 Data Description

The range of different socio-economic variables that may be used as indicators of energy demand are listed in Table 1. These range from indicators that are common across sectors (such as employees or gross value added) to sector specific indicators (such as the hectare of farm land or the distance travelled by vehicles).

**Table 1:** Local energy usage indicators by sector and region; Ireland, South West, Kerry and the Dingle peninsula.

Sector	Indicator	Unit	Ireland	South West	Kerry	Dingle
General	Population	-	4,761,865	564,918	147,707	12,508
Agriculture & Fishing*	Employees	-	89,116	16,859	4,913	646
Industry	Employees	-	210,059	40,331	5,695	433
Commercial Services	Employees	-	1,197,382	129,939	25,471	3,899
Public Services	Employees	-	460,358	46,028	8,657	711
Total	Employees	-	1,956,915	233,157	44,736	5,689
Agriculture	Gross value added	€ million	€2,183	€451	€125	-
Fishing	Gross value added	€ million	€372	€171	€28	€24
Industry	Gross value added	€ million	€94,485	€31,252	€1,314	-
Commercial Services	Gross value added	€ million	€127,741	€15,373	-	-
Public Services	Gross value added	€ million	€29,229	€3,951	-	-
Total	Gross value added	€ million	€254,011	€51,198	-	-
Industry	Buildings	-	15,996	2,550	561	-
Commercial Services	Buildings	-	263,676	28,869	8,535	-
Public Services	Buildings	-	33,473	3,865	1,065	-
Residential	Buildings	-	1,697,665	200,340	54,288	4,834
Agriculture	Area of land	hectare	4,991,353	909,043	347,241	33,748
Fishing	Unladen weight of boats	Tonne	63,964	17,087	2,856	-
Transport - Road freight	Vehicles	-	338,431	52,516	12,063	-
Transport - Private car	Vehicles	-	2,038,268	318,719	69,512	6,845
Transport - Public service	Vehicles	-	31,516	3,716	959	
Transport - Road freight	Distance travelled	million km	7,750	1,229	261	
Transport - Private car	Distance travelled	million km	36,689	5,806	1,302	128
Transport - Road freight	Weight carried	Tonne km	11,564	1,615		

In the majority of cases, these variables came from the 2016 Census of Ireland. Thus, the same year was taken for the national energy balance, the breakdown per sector in 2016 is shown in Table 2.

**Table 2:** Energy demand by sector in Ireland 2016.

Sector	GWh
Agriculture & Fishing	
Agriculture incl. forestry	2,407
Fisheries	221
Industry	28,435
Residential	31,448
Services	
Commercial Services	9,688
Public Services	6,094
Transport	
Road freight	12,255
Private car	24,970
Public Services	1,553
Unspecified	4,488
Total	121,559

Combining the data from Tables 1 & 2, we can produce a list of unit energy consumption values, as displayed below in Table 3. The unit energy consumption is the amount of energy demand per statistical indicator. The values shown here are based on data from 2016 as that is the year of the most recent Census. It provides a useful snapshot of the energy demand in the various sectors. However, as discussed in McGookin et al. [1], could be improved with access to more detailed data that would allow an assessment of the changes over time and also regional variations within the country. Prior to the impact of the COVID-19 pandemic, Ireland's energy demand had remained fairly stagnate over the period 2016–2019 due to limited progress addressing energy-related CO2 emissions in heating and transport [2], thus the variation over that period to values shown here may be minor.

**Table 3:** Unit energy consumption by sector and indicator for Ireland in 2016.

Sector	Indicator	Unit	Unit Energy Consumption
Agriculture & Fishing	Population weighted	MWh / capita	0.552
Agriculture & Fishing	No. of employees	MWh / employee	29.494
Agriculture	Gross value added	GWh / €mnGVA	1.103
Agriculture	Area of land	MWh / hectare	0.482
Fishing	Gross value added	GWh / €mnGVA	0.594
Fishing	Unladen weight of boats	MWh / tonne	3.455
Industry	Population weighted	MWh / capita	5.971
Industry	No. of buildings	GWh / building	1.778
Industry	No. of employees	MWh / employee	135.369
Industry	Gross value added	MWh / €mnGVA	300.95
Residential	Population weighted	MWh / person	6.604
Residential	No. of homes	MWh / house	18.524
Residential	No. of homes adjusted by type	MWh / house	17.735
Services	Population weighted	MWh / capita	3.314
Commercial Services	No. of buildings	MWh / building	36.741
Commercial Services	No. of employees	MWh / employee	8.091
Commercial Services	Gross value added	MWh / €mnGVA	75.839
Public Services	No. of buildings	MWh / building	182.061
Public Services	No. of employees	MWh / employee	13.238
Public Services	Gross value added	MWh / €mnGVA	208.493
Transport	Population weighted	MWh / capita	9.086
Transport - Road freight	No. of vehicles	MWh / vehicle	36.210
Transport - Road freight	km travelled	GWh / million km	1.581
Transport - Road freight	Tonne km	GWh / mil tonne km	1.060
Transport - Road freight	Gross value added	MWh / €mnGVA	48.245
Transport - Private car	No. of vehicles	MWh / vehicle	12.250
Transport - Private car	km travelled	MWh / million km	680.577
Transport - Public Services	No. of vehicles	MWh / vehicle	49.289

The breakdown of energy supply per fuel at a national level is displayed in Table 4. In addition, moving to a more granular detail, the primary heating fuel in non-domestic buildings is provided by the BER database for both Ireland and County Kerry in Table 5. The residential heating fuel choice from the 2016 Census is available for the area of interest, the Dingle Peninsula, in Table 6. Finally, Table 7 provides values for the average size of households and annual average energy demand per metre by the year of construction. This is based on the values recorded during building energy rating (BER) assessments carried out from 2009 up to the end of 2016.

**Table 4:** Energy supply per sector in Ireland in 2016.

	Oil	Natural Gas	Coal	Peat	Grid Electricity*	Petrol	Diesel	RES-H	RES-E*	RES-T
Agriculture & Fishing										
Agriculture incl. forestry					23.2%		76.8%			
Fisheries							100%			
Industry	20%	32.9%	4.5%		35.3%			7.3%		
Residential	37.2%	20.8%	7%	7%	25%			1.9%	1.2%	
Services										
Commercial Services	19.0%	24.2%			51.5%			1.9%	3.3%	
Public Services	17.0%	49.4%			32.3%			0.5%	0.8%	
Transport										
Road freight					0.02%	0.2%	92.5%			7.2%
Private car					0.1%	49.0%	44.6%			6.3%
Public Services					0.05%	18.3%	74.7%			6.9%
Unspecified					0.02%	28.2%	65.1%			6.7%

*Note: In 2016, 27.2% of the grid electricity came from renewable sources. The RES-E shown in this table represents only on-site small-scale generation such as solar PV.

**Table 5:** Primary heating fuel in non-domestic buildings Ireland and Co. Kerry in 2016.

	Mains Gas	Oil	LPG	Electricity	Solid Fuel
National	25%	12%	2%	62%	1%
Kerry	0%	24%	6%	69%	1%

**Table 6:** Primary central heating fuel for households on the Dingle Peninsula in 2016.

	Solid Fuel		Oil		RES-H		
Fuel Type	Peat	Coal	Kerosene	LPG	Renewable	Wood	Electricity
No. of houses	277	524	3290	156	66	199	322
	801		3446		265		322

% share	5.7%	10.8%	68.1%	3.2%	1.4%	4.1%	6.7%
	16.6%		71.3%		5.5%		6.7%

**Table 7:** Average annual energy demand per metre squared and floor area of Irish households by year of construction

Period Built	kWh/m^2^/year	Avg·m^2^
Pre 1919	402	118
1919–1945	409	100
1946–1960	365	104
1961–1970	328	104
1971–1980	269	109
1981–1990	240	108
1991–2000	225	110
2001–2010	187	113
2011 or later	78	140
Not stated	266	122

## 2 Experimental Design, Materials and Methods

This section outlines the steps taken to acquire the data contained within this article.

### 2.1 Data handling process

**Fig. 1:**
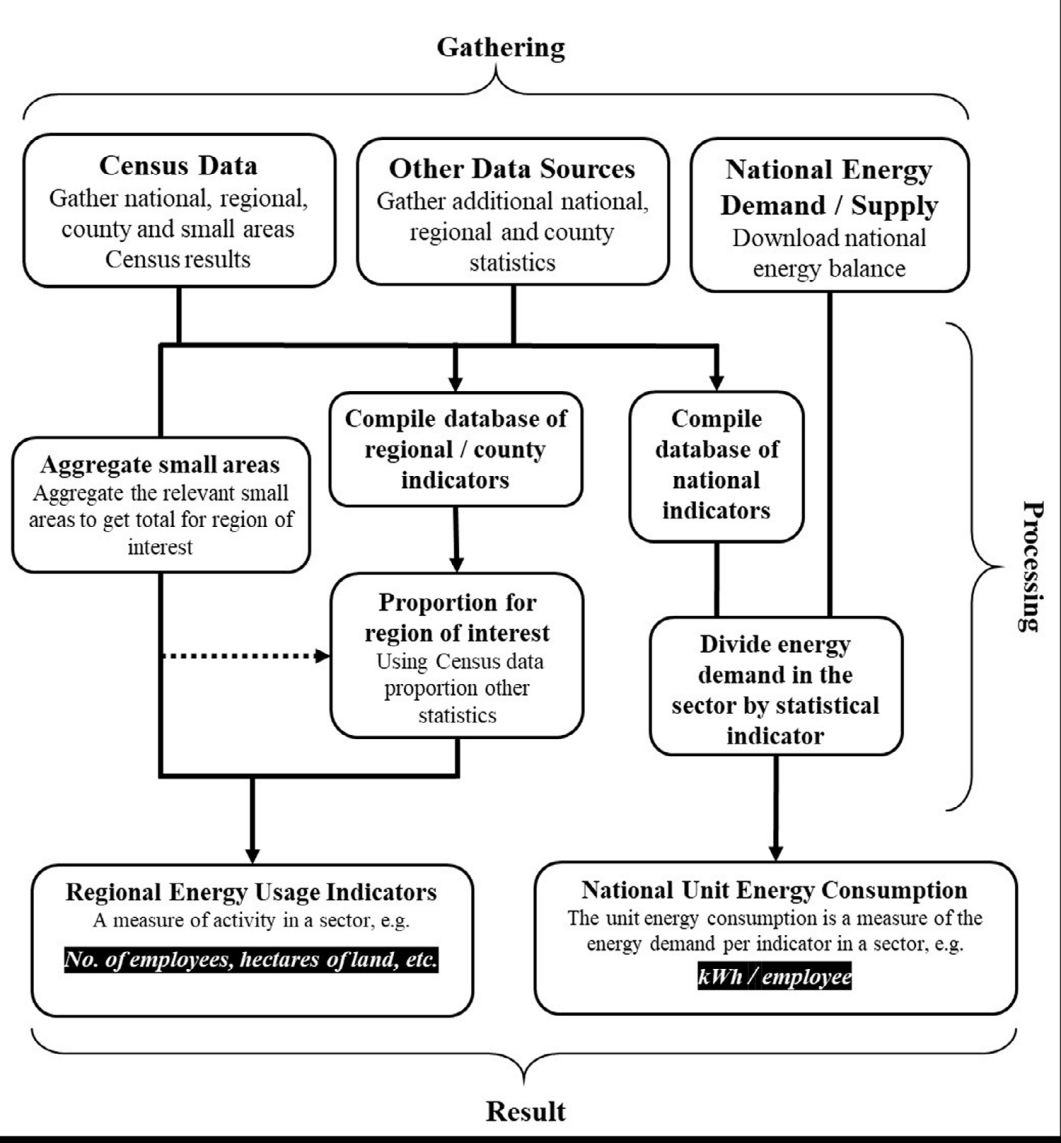
Flowchart of the data handling process.

A flowchart of the primary data gathering process is provided in Fig. 1. There are two central parts. Firstly, the gathering of regional energy usage indicators by aggregating census data for the small areas that make up the case study region (see Section 2.2) or proportioning other key socio-economic variables that we only available at the county or NUTS 3 regional level (see Section 2.3). Secondly, the creation of national unit energy consumption values using national energy usage indicators combined with the energy demand in each sector.

### 2.2 Census data

The data collected as part of the Census of Ireland is available for download as a comma separate file from the Central Statistics Office (CSO) repository [3], along with a glossary to navigate the dataset. It can also be viewed through an interactive tool called ‘SAPMAP’, which stands for Small Area Population Map [4]. The survey data is aggregated for a range of spatial levels from the National down to County level of which there are 31 in Ireland and finally the Small Area of which there are 18,641. It contains 45 tables organised according to the themes of the Census survey. The first step of filtering was to identify the relevant Small Areas using the SAPMAP tool. The 24 Small Areas that make up the Dingle Peninsula are listed in Table 8, along with the GEOGID that was used to find them in the comma-separated file downloaded. In addition, the three electoral districts that make up the South West region are also listed.

**Table 8:** List of the Small Areas extracted from the CSO database.

GEOGID	Name	Region
ED3409_19026	Ballinvoher	Dingle Peninsula
ED3409_19028	Ballynacourty	
ED3409_19037	Inch	
ED3409_19034	Dún Chaoin	
ED3409_19035	Dún Urlann	
ED3409_19038	Cill Maoilchéadair	
ED3409_19039	Cill Chuáin	
ED3409_19042	Márthain	
ED3409_19137	Baurtregaum	
ED3409_19138	Blennerville	
ED3409_19152	Kilgobban	
ED3409_19156	Knockglass	
ED3409_19030	Castlegregory	
ED3409_19032	Deelis	
ED3409_19044	An Sráidbhaile	
ED3409_19027	An Baile Dubh	
ED3409_19041	Lack	
ED3409_19139	Boolteens	
ED3409_19151	Kilgarrylander	
ED3409_19029	Cé Bhréanainn	
ED3409_19031	An Clochán	
ED3409_19033	An Daingean	
ED3409_19036	Na Gleannta	
ED3409_19045	Ceann Trá	

CTY31_CC	Cork County	South West
CTY31_CK	Cork City	
CTY31_KY	Kerry	

Having extracted the data for the relevant Small Areas, the second filtering step involved identifying the relevant tables and variables using the glossary of terms, as listed in Table 9.

**Table 9:** List of the Census tables used by theme, data provided, reference name in dataset and short description.

Theme	Table Name	Column Name	Description
Theme 1: Sex, Age and Marital Status	Table 1 Population aged 0–19 by sex and year of age, persons aged 20 years and over by sex and age group	T1_1AGETT	Total population
Theme 6: Housing	Table 2 Permanent private households by year built	T6_2_PRE19H	Pre 1919 (No. of households)
		T6_2_19_45H	1919–1945 (No. of households)
		T6_2_46_60H	1946–1960 (No. of households)
		T6_2_61_70H	1961–1970 (No. of households)
		T6_2_71_80H	1971–1980 (No. of households)
		T6_2_81_90H	1981–1990 (No. of households)
		T6_2_91_00H	1991–2000 (No. of households)
		T6_2_01_10H	2001–2010 (No. of households)
		T6_2_11LH	2011 or Later (No. of households)
		T6_2_NSH	Not stated (No. of households)
		T6_2_TH	Total (No. of households)
Theme 6: Housing	Table 5 Permanent private households by central heating	T6_5_NCH	No central heating
		T6_5_OCH	Oil
		T6_5_NGCH	Natural gas
		T6_5_ECH	Electricity
		T6_5_CCH	Coal (incl. anthracite)
		T6_5_PCH	Peat (incl. turf)
		T6_5_LPGCH	Liquid petroleum gas (LPG)
		T6_5_WCH	Wood (incl. wood pellets)
		T6_5_OTH	Other
		T6_5_NS	Not stated
		T6_5_T	Total
Theme 14: Industries	Table 1 Persons at work by industry and sex	T14_1_AFFT	Agriculture, forestry and fishing - Total
		T14_1_BCT	Building and construction - Total
		T14_1_MIT	Manufacturing industries - Total
		T14_1_CTT	Commerce and trade - Total
		T14_1_TCT	Transport and communications - Total
		T14_1_PAT	Public administration - Total
		T14_1_PST	Professional services - Total
		T14_1_OTHT	Other - Total
		T14_1_TT	Total
Theme 15: Motor Car Availability, PC Ownership and Internet Access	Table 1 Number of households with cars	T15_1_NC	No motor car
		T15_1_1C	1 motor car
		T15_1_2C	2 motor cars
		T15_1_3C	3 motor cars
		T15_1_GE4C	4 or more motor cars
		T15_1_NSC	Not stated
		T15_1_TC	Total

As this data came from a Census survey, there was some processing required. For age of households, central heating systems and the number of cars, if respondents had chosen “*not stated*”, an average value was used. In addition, the central heating system data required the following restructuring;

• No central heating was taken to be solid fuel, split between peat and coal
• Natural gas was assumed to be LPG as there is no gas grid in Kerry

### 2.3 Other sources of energy usage indicators

There were a number of additional sources used to get sector specific indicators. These are listed in Table 10 below, along with a brief description of the necessary processing.

**Table 10:** List of socio-economic indicators and sources.

Sector	Indicator	Source	Granularity	Processing
Agriculture	Hectares of land	Census of Agriculture [5]	Available by Small Area	Aggregate values from relevant Small Areas, as per Table 7
Agriculture	Gross value added	CSO County Income and Regional GDP 2016 [6]	Average farming income reported per County	South West GVA in 2016 split between Kerry and Cork based on the share of farming incomes from 2010 Census of Agriculture [5]
Fishing	Gross value added	CSO Fish Landings 2007-2018 [7]	Reported by port, i.e. area of interest (Dingle)	None
Fishing	Unladen weight of boats	Irish Sea Fishing Fleet Register [8]	County level registration	Weight of the boats registered to each county reported annually
Industry	Employees	CSO Business Demography [9]	County level reporting	None
Industry	Gross value added	CSO County Income and Regional GDP 2016 [6]	Regionally reported Ireland South West (Kerry and Cork)	South West GVA in 2016 split between Kerry and Cork based on historic share [10]
Industry &Services	Enterprises	CSO Business Demography [9]	County level reporting	None
Residential	Energy per house	The Irish Commission for Energy Regulation (CER) ‘*Review of Typical Domestic Consumption Values for Electricity and Gas Customers*’ [11]	Average values for heating and electricity per household by type (one-off or terraced)	The share of one-off rural housing in Co. Kerry is 74.1% [12], number of houses in Dingle Peninsula provided by Census data
Services	Gross value added	CSO County Income and Regional GDP 2016 [6]	Regionally reported Ireland South West (Kerry and Cork)	None
Services	Employees	CSO Business Demography [9]	County level reporting	‘Other’ category listed in Table 8 split between public / commercial based on a population-based proportioned of public sector employees from the county level
Transport	Vehicles	Irish Bulletin of Vehicle and Driver Statistics 2016 [13]	County level reporting	None
Transport - Road freight & Private car	Distance travelled	CSO Transport Omnibus 2016; Road traffic volumes [14]	County level reporting	None
Transport - Road freight	Weight carried	CSO Transport Omnibus 2016; Road freight transport [15]	Regional level reporting	None

### 2.4 National energy demand and energy supply by sector

The national energy balance from 1990–2019 can be downloaded as an Excel file from the Sustainable Energy Authority of Ireland's (SEAI) [16]. It is given in Ktoe and was converted to GWh, using the conversion 1 Ktoe is 11.63 GWh.

### 2.5 Building energy rating database

The share of heating fuels in non-domestic buildings is taken from the Irish energy performance certificate database known as the Building Energy Rating or BER, it is reported quarterly through the CSO [17]. Similarly, the residential figures are also reported quarterly [18]. This provides figures for the average size (m^2^) of houses in Ireland by age and the associated energy demand per metre squared (kWh/m^2^).

## Ethics Statement

Not applicable.

## CRediT Author Statement

**Connor McGookin**: Conceptualization, Methodology, Formal analysis, Investigation, Writing original draft, Visualization; **Brian O’ Gallachoir**: Conceptualization, Methodology, Supervision, Writing review & editing; **Edmond Byrne**: Conceptualization, Methodology, Supervision, Writing review & editing.

## Declaration of Competing Interest

The authors declare that they have no known competing financial interests or personal relationships which have or could be perceived to have influenced the work reported in this article.
